# A Few Points in the Examination of Mental Cases

**Published:** 1908-10-24

**Authors:** 


### Mental Diseases.

### A FEW POINTS IN THE EXAMINATION OF MENTAL CASES.

In cases of mental excitement or depression but little difficulty is experienced in obtaining facts sufficient for certification, but in certain cases the examination may be a very difficult proceeding, especially where the patient is suspicious of strangers or has some idea of the object of the visit. The patient who is aware that other people believe him to be deluded and are anxious to place him under restraint is capable of keeping his delusions for a time in the background, or of so "hedging" to questions that much skill and patience are required to discover his true mental state. Information as to the patient's conversation and behaviour are readily supplied by others, but as facts observed by the medical man "at the time of examination" are essential for certification, it is essential that the points to be observed should be kept in view, and that some method should be used to extract them.

When called to examine a patient who is a stranger, it is as well to inquire first from the attendant practitioner or from the friends as to the temperament, tastes, temper, intelligence, etc., of the individual when sane, as a knowledge of these points is necessary for correctly judging departure from the normal. During examination information is to be obtained on many points, and this only by more or less prolonged conversation on ordinary topics; the search for delusions, which the patient is on his guard not to mention, being left for some time, that his reserve may be broken. A physical examination, even if unnecessary, is often useful to open a way to conversation, but the best manner of commencing will depend on the mental attitude of the patient to the doctor and on the tact and resource of the latter.

The most important rules to be observed in the conversation are, to let the patient do as much of the talking as possible, and to avoid leading questions. The topics do not matter; they should at first have no connection with the known delusions, but should gradually be led round to them. The power of attention should be especially noticed, as lack of this power, to the ordinary extent, is one of the earliest symptoms of mental unsoundness. Sullen, suspicious, restless or impulsive behaviour, laughter without discoverable cause, easy loss of self-control, and other abnormalities of conduct, may give warning of hallucinations of some special sense or of delusions. Deficiency of the general intelligence, as compared with the patient's normal standard, or with that which would be expected from his degree of education, may be noted. Incoherence in any marked degree is at once apparent, but the manner of thought and association require more careful observation. The emotions are important. Are they called into play with abnormal ease? Do they seem to rule the conduct unduly? Is antipathy displayed to any particular person or persons without sound reason? In the answer to this last question will sometimes be found the clue to delusions. Memory for recent events is lost sometimes, particularly in alcoholic dementia, whilst that for more remote events is deficient in other cases.

Abnormal consciousness of self is a striking trait in melancholic patients, in the grandiose delusions of general paralysis of the insane, in alcoholics, and in the self-satisfied egoism of some other conditions. Power of reasoning and judgment may be seriously deficient either generally or on some special subjects, the patient being, so far as these are concerned, unable to distinguish between the essential and the non-essential or trivial. The imagination, too, and will power can both be tested.

The above points can be noted in the course of an ordinary conversation, and information on them forms valuable evidence as regards sanity or otherwise. If hallucinations or delusions are suspected or known to exist, but have not been brought out, questions as to their existence may be put. These direct questions are seldom necessary, however, as the patient will rarely talk for long without mentioning them if they do exist. Should the patient make statements which are believed to be delusions, but which may possibly be true or merely errors due to misunderstanding, care should be taken to ascertain that they really *are* delusions before stating the fact on a certificate. This, though generally easy, is, unfortunately, not always done.

